# RNA sequencing of early round goby embryos reveals that maternal experiences can shape the maternal RNA contribution in a wild vertebrate

**DOI:** 10.1186/s12862-018-1132-2

**Published:** 2018-03-22

**Authors:** Irene Adrian-Kalchhauser, Jean-Claude Walser, Michaela Schwaiger, Patricia Burkhardt-Holm

**Affiliations:** 1 Program Man-Society-Environment, Department of Environmental Sciences, University of Basel, Vesalgasse 1, CH-4051 Basel, Switzerland; 2 Department of Environmental Systems Science, Genetic Diversity Centre Zurich, ETH Zurich, Universitätstrasse 16, CH-8092 Zurich, Switzerland

**Keywords:** parental effects, non-genetic inheritance, short term adaptation, maternal contribution, *Neogobius melanostomus*

## Abstract

**Background:**

It has been proposed that non-genetic inheritance could promote species fitness. Non-genetic inheritance could allow offspring to benefit from the experience of their parents, and could advocate pre-adaptation to prevailing and potentially selective conditions. Indeed, adaptive parental effects have been modeled and observed, but the molecular mechanisms behind them are far from understood.

**Results:**

In the present study, we investigated whether maternal RNA can carry information about environmental conditions experienced by the mother in a wild vertebrate. Maternal RNA directs the development of the early embryo in many non-mammalian vertebrates and invertebrates. However, it is not known whether vertebrate maternal RNA integrates information about the parental environment. We sequenced the maternal RNA contribution from a model that we expected to rely on parental effects: the invasive benthic fish species *Neogobius melanostomus* (Round Goby). We found that maternal RNA expression levels correlated with the water temperature experienced by the mother before oviposition, and identified temperature-responsive gene groups such as core nucleosome components or the microtubule cytoskeleton.

**Conclusions:**

Our findings suggest that the maternal RNA contribution may incorporate environmental information. Maternal RNA should therefore be considered a potentially relevant pathway for non-genetic inheritance. Also, the ability of a species to integrate environmental information in the maternal RNA contribution could potentially contribute to species fitness and may also play a role in extraordinary adaptive success stories of invasive species such as the round goby.

**Electronic supplementary material:**

The online version of this article (10.1186/s12862-018-1132-2) contains supplementary material, which is available to authorized users.

## Significance statement

In wild species, the extent to which parents are able to provide environmental information may determine the fitness of their offspring. This implies that the ability to provide parental information may be relevant for the survival and adaptation of species. Surprisingly, however, we know little about non-genetic inheritance in wild species.

Maternal genes direct early development in many animals. We studied the maternal RNA contribution in a wild fish species and found that the composition of maternally contributed RNA reflected the temperature experienced by the mother. Our findings indicate that maternally inherited RNA may not only encode a developmental start-up kit, but may also contain vital information about the environmental conditions.

## Background

It has been proposed that species who live in fluctuating environments have a fitness advantage if they can draw from the experience of their parents [20, 33]. Non-genetic inheritance has been proposed to constitute a potentially important, yet poorly understood element in the response of populations to environmental change and fluctuations [7]. According to models, parental effects [57] are expected to evolve when selection is strong early in life, when parental and offspring environments positively correlate, and when opportunities for genetic adaptation are limited [20, 33, 34]. Indeed, adaptive parental effects have been observed in plants (e.g. [22]) and several vertebrate taxa (e.g. [56]), including humans (e.g. [42]). To our present knowledge, parental effects may be mediated by proteins, hormones, lipids, messenger RNAs, noncoding RNAs, histone modifications or DNA methylation patterns. Otherwise, we know very little about parental effects, about their prevalence, and about their role for adaptive processes in wild populations. It is unclear whether parental effects are common or rare in natural settings, we do not know which molecular mechanisms are relevant in wild vertebrates, and it has not been assessed systematically which types of information can be conveyed to the next generation.

The maternal RNA contribution plays a crucial role during early development in a wide range of organisms. In many species, development of the early embryo is directed exclusively by maternal factors [18]. For example, fruit fly embryonic polarity and segmentation [11, 54], amphibian or fish development up to the 64- to 128-cell stages [2] and the initiation of mammalian development [69] depend on so-called maternal or maternal-effect genes. The embryonic genome is transcriptionally quiescent and therefore does not contribute to development until the Maternal-Zygotic Transition (MZT; [59]). In fish, this transition from maternal to embryonic control takes place after the 64-cell stage [1, 26, 27, 35, 43]. Up to the MZT, maternally contributed RNA is mostly stable [5, 6, 17, 47]. At the MZT, the embryo initiates transcription [59] and targeted and controlled clearing of 30–50% of maternal messages [6, 63]. Maternal genes, however, still amount to 60–70% of mRNA molecules at the peak of zygotic expression in zebrafish [35] and are required for the correct execution of morphogenetic steps long after the MZT [62].

It is currently unclear whether maternally contributed RNA provides information beyond how to physically construct an organism in wild species and under natural conditions (for example, on how to finetune its energy metabolism, or its behaviour). Most research on maternal RNA focuses on developmental roles of maternal RNA or on mechanistic aspects of RNA regulation and stability in model organisms such as C.elegans [40], Xenopus [48], or the fruit fly [46, 54, 55]. Screens in these organisms have uncovered maternal genes with roles in morphogenesis and cell fate determination. They were however not designed to detect maternal genes inducing subtle or condition-dependent changes in morphology, metabolism, or behaviour. Clues that indicate a more subtle role for maternal RNA come from fertility medicine and fish aquaculture. When ovulation is forced by light regime manipulation or hormone administration in rainbow trout, the levels of a small subset of messages differ from natural ovulation. These subtle changes are related to a decreased developmental potential of eggs from induced ovulations [8]. Similarly, ovary transcriptome profiles from striped bass indicated a major effect of small changes in transcriptome composition on egg developmental potential [12]. These findings suggest that small variations in the levels of maternally contributed RNAs can have profound effects on embryo development, survival and fitness.

Following a mechanistic approach [42, 57], we therefore aimed to investigate whether the maternal RNA contribution was capable to reflect a maternal experience in a natural population of vertebrates. To this end, we used a vertebrate model that we expected to rely on parental effects based on its life history traits and ecological properties [20, 33]. *Neogobius melanostomus* (the round goby) is a benthic fish species which deals with novel environments remarkably well. It is invasive in many parts of Europe and Northern America. In the sampled population, round goby females are sexually mature at age one or two. They reach an average age of two to three years, and a maximum age of four years [60, 28]. Round goby females can spawn up to six times per summer once the water temperature reaches 10–11 °C, but in the sampled population, two to three batches are more common ([60, 29]). Embryonic development takes between 14 and 20 days [13]. Maternal investments in egg provisions are large [39], and females adapt reproductive effort flexibly to environmental conditions and disturbances [29]. Finally, number of offspring is high (up to 5000 eggs per female, [39]) and selection pressure peaks during early life stages (survival to age = 1 is less than 1%, [32]). Thus, the round goby meets the modeled requirements for the evolution of parental effects [34]. The home range of round goby is limited [50], and site fidelity is strong [67], with 90% of females recaptured at the same location after one year when using traps spaced 25 m apart [38]. Therefore, individuals from one site share a common history in terms of environmental conditions.

To test whether the maternal contribution reflected maternal experiences in the round goby, we collected early cleavage embryos from a wild *N. melanostomus* population at water temperatures from 14 to 25°C. We selected fourteen samples at the 32 cell stage or younger. We sequenced and assembled the maternal RNA contribution from these embryos and compared it against publicly available data from zebrafish and against the round goby draft genome. We confirmed that maternal RNA is stable up to the 32 cell stage in round goby, and then determined whether the water temperature experienced by the mother was reflected in the maternal RNA by PCA and correlation analyses. Finally, we identified individual temperature-responsive maternal genes and pathways.

## Results

### Embryonic development

**Fig. 1:**
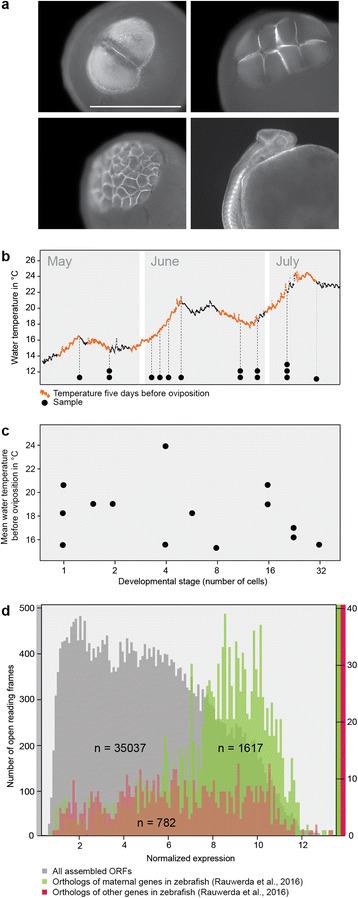
Sampling, sequencing and verification of the *Neogobius melanostomus* maternal RNA contribution. **a**, Visualization of embryonic development by actin staining. 2-cell, 8-cell, > 32-cell, and organogenesis stage are depicted. Scale bar: 1 mm. See Additional file 1: Figure S1 for a full panel of developmental stages. **b**, Sampling. The ascending line represents water temperature at the sampling site. Dashed lines and dots indicate sampling dates and samples, respectively. The temperature curve during five days before each sampling date is colored orange. **c**, Developmental stage of sequenced samples in relation to the mean water temperature during five days before oviposition. Each dot represents a sample. **d**, The round goby orthologs of maternally contributed genes in the zebrafish [49] are highly expressed in pre-MZT *N. melanostomus* embryos

We morphologically characterized round goby embryonic development up to organogenesis using phalloidin stainings of the actin cytoskeleton (Fig. 1a, Additional file 1: Figure S1). We found that cleavage divisions of *N. melanostomus* are overall similar to zebrafish [31]. From the 2- to the 4-cell stage, cell division happens at an oblique angle, which persists up to the 8-cell stage (Additional file 1: Figure S1). The inner cells of the embryo lose contact with the yolk and form a second layer of cells at the late 32-cell stage. Since the embryo is smaller relative to yolk volume than in zebrafish, and since neither embryo nor yolk are transparent, “high”, “oblong” or “sphere” stages (which refer to the overall shape of embryo and underlying yolk) are difficult to distinguish in *N. melanostomus*. Overall, however, embryonic development follows established patterns (Additional file 1: Figure S1; [31]).

### Sequencing and assembly of maternal RNA

We sequenced and assembled the maternal RNA from 14 pre-MZT embryos and from one prim stage sample harvested at water temperatures from 14 to 24°C (Fig. 1b, c). De-novo assembly yielded 46,560 open reading frames (ORFs), of which 35,037 were expressed in early embryos and matched gene models for round goby (Additional file 2: Table S1; Additional file 3: Data S1; unpublished genome sequence). We compared the expression of the assembled ORFs with maternal RNA expression data from zebrafish [49] and found that maternally expressed zebrafish genes were also highly expressed in pre-MZT round goby embryos (Fig. 1d).

### Effect of maternal temperature experience on the maternal RNA contribution

Next, we tested whether maternal RNA expression was related to cleavage stage (which would indicate that MZT happens before the 64-cell stage in round goby), to genetic distance (which has previously been shown to impact the expression of a subset of maternal genes in zebrafish; [49]), or to maternal temperature experience in the last five days before oviposition. Cleavage stage and genetic dissimilarity were used to determine the baseline or chance level of correlation between a given parameter and gene expression. Both parameters provide a buildt-in control for false discovery rates. This means that a maternally experienced parameter would have to affect the transcriptome to a greater extent than either cleavage stage or genetic dissimilarity to be considered significant. The testing variables were mean, minimum, and maximum temperature experienced by the mother during five days before oviposition, the average temperature on the sampling day, and the temperature interval experienced by the mother during five days before oviposition. These parameters were calculated from water temperature information logged continuously at the egg collection site (Fig. 1b, Additional file 4: Figure S2).

**Fig. 2:**
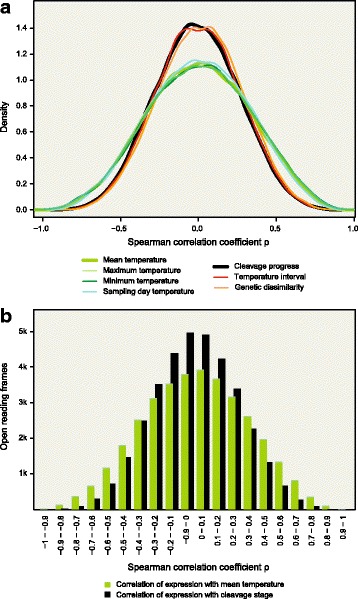
The expression of maternally contributed RNAs correlates with maternally experienced mean, maximum, and minimum temperature, but not with control parameters. **a**, Density plot of the Spearman correlation coefficients calculated between expression level and indicated parameters. **b**, Barplot showing the number of open reading frames that correlate with mean temperature experienced by the mother before oviposition (green) or with cleavage stage (black) at the indicated correlation values

To test for a link between maternal experience and maternal RNA expression, we performed correlation analysis and PCA analysis of expression levels versus control parameters and testing parameters. Using rank based models, we quantified the correlation between the expression of each open reading frame and each of the parameters. We found that expression correlated only weakly with temperature interval, genetics, or cleavage stage, indicating that these parameters captured the baseline correlation levels expected by chance. Expression levels were correlated with the mean, minimum, or maximum temperature experienced by the mother before oviposition above the baseline levels established by the control parameters (Fig. 2, Tables 1 and 2).

**Table 1:** Correlation analysis

	ORFs correlating with			
	mean temperature	cleavage progress	temperature interval	genetic dissimilarity
correlating at *p* < = 0.01	707	161	144	131
correlating at *p* < = 0.05	1894	725	710	679
anticorrelating at *p* < = 0.01	2076	817	751	850
anticorrelating at *p* < = 0.05	741	177	151	181

Number of open reading frames correlating with the indicated parameters at the indicated *p*-values

**Table 2:** Correlation analysis

	ORFs correlating with			
Spearman’s rho	mean temperature	cleavage progress	temperature interval	genetic dissimilarity
> = 0.9	6	0	1	0
> = 0.8	99	6	9	7
> = 0.7	457	87	70	83
> = 0.6	1268	358	336	398
> = 0.5	2605	1020	1003	1137
> = 0.4	4572	2334	2390	2701
> = 0.3	7183	4592	4778	5227
> = 0.2	10,352	7973	8111	8843
> = 0.1	14,016	12,209	12,553	13,363
> = 0.0	17,944	17,118	17,445	18,311
<=0.0	17,174	18,024	17,693	16,828
<= − 0.1	13,296	12,958	12,819	11,920
<= − 0.2	9764	8573	8268	7719
<= − 0.3	6637	5066	4837	4423
<= − 0.4	4116	2574	2449	2265
<= − 0.5	2317	1110	1052	941
<= − 0.6	1157	388	349	305
<= − 0.7	503	96	83	57
<= − 0.8	143	16	7	5
<= − 0.9	20	0	0	0

Number of open reading frames correlating with the indicated parameters with the indicated correlation coefficient

**Fig. 3:**
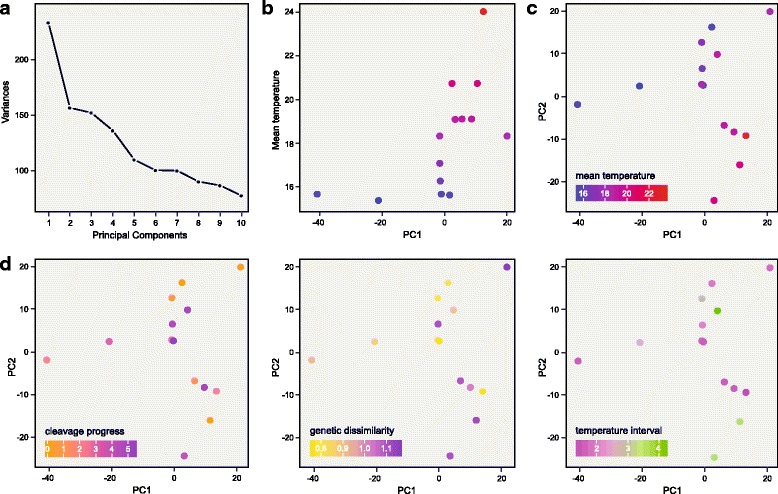
The maternal temperature experience before oviposition has a global effect on the maternal RNA contribution. **a**, Variance explained by individual principal components. **b**, PC1 correlates with the mean temperature experienced by the mother. **c**, PC1 and PC2 together explain mean temperature experienced by the mother. **d**, Cleavage progress, genetic dissimilarity, or temperature interval experienced by the mother are independent from PC1 and PC2

PCA analysis further confirmed that the expression of maternal RNA was related to maternally experienced temperature. PC1, which explained the largest proportion of the variance, correlated with mean maternally experienced temperature (Fig. 3a and b). Together, PC1 and PC2 isolated a cluster of samples which we found to correspond to the samples with highest mean temperature (Fig. 3c). Cleavage stage, genetic distance, or maternally experienced temperature interval were not related to PC1 or PC2 (Fig. 3d), or to any other principal component (Additional file 5: Figure S3). Biplots between samples, and biplots with correlating genes or PCA loading genes marked, as well as a comparison of correlation values and PCA loading are available as supplementary information (Additional file 6: Data S2).

### Temperature-effect on individual genes and pathways

**Fig. 4:**
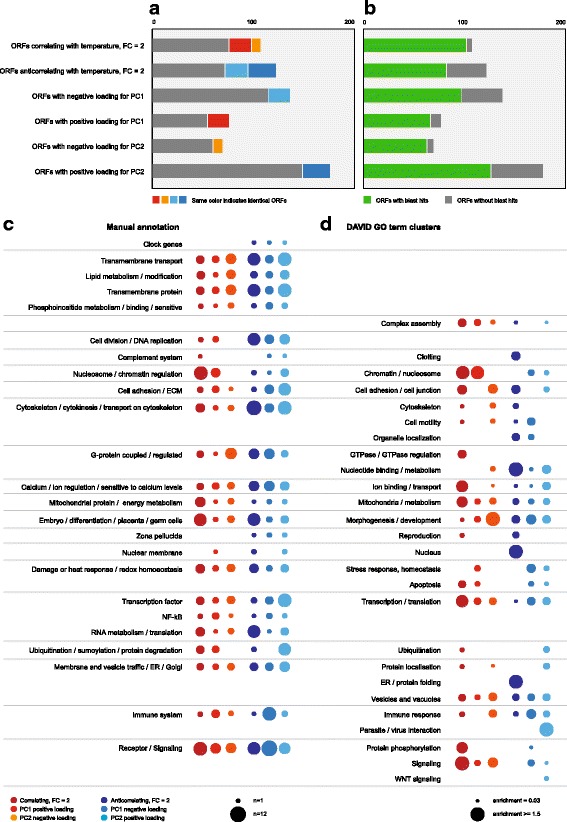
Functional analysis of the temperature response. **a**, Correlation analysis and PCA identify overlapping sets of genes. **b**, Open reading frames which react to higher temperature with increased expression are more conserved. **c**, Pathways and functions of temperature sensitive maternal genes, manual annotation. Dot size corresponds to the number of genes with that function. **d**, GO term clusters identified among temperature sensitive maternal genes by the DAVID annotation tool. Dot size corresponds to DAVID enrichment score

We then evaluated the functional impact of the maternal temperature experience on maternal RNA composition. To this end, we focused on ORFs displaying a two-fold or higher change in expression in response to temperature according to correlation analyses (*n* = 120 for positive and *n* = 105 for negative correlation with mean experienced temperature), and on ORFs with above-average positive or negative loadings (contributions) for PC1 (*n* = 136 / 75) or PC2 (*n* = 176 / 68). As expected, we found that these ORF sets were partially overlapping. ORFs with negative loadings for PC1 or positive loadings for PC2 overlapped with temperature-correlating ORFs, while ORFs with negative loadings for PC1 and positive loadings for PC2 overlapped with temperature-anticorrelating ORFs (Fig. 4a; Additional file 4: Data S1). We blasted the selected ORFs to identify human orthologues for functional annotation. Interestingly, warmth-responsive ORFs (represented by correlating ORFs, ORFs with positive loading for PC1, and ORFs with negative loading for PC2) were more likely to produce blast hits than cold-responsive ORFs (represented by anticorrelating ORFs, ORFs with negative loading for PC1, and ORFs with positive loading for PC2; Fig. 4b).

We then performed functional analyses based on gene information available on NCBI (Additional file 7: Table S2). Round goby are poikilotherm organisms. Their metabolic rates and developmental pace depends on ambient temperature. Accordingly, we expected to find genes related to development, to energy metabolism, or to components of the stress- or heat shock response among temperature-responsive ORFs. Indeed, we identified 15 mitochondrial / energy metabolism-related proteins. We also identified two genes with heat shock pathway annotations, two clock gene orthologues (CIART and PER2), and 12 genes involved in stress response, DNA repair, or redox homeostasis. In addition, the list contained several ion- and calcium-sensitive processes or proteins, many cytoskeletal components and proteins involved in cell adhesion, GTPases and G-protein-related processes, as well as pathways related to lipid synthesis or modification and membrane function. We also identified signaling proteins and proteins involved in transcriptional regulation (for example, four components of the Notch signaling pathway), and, to our surprise, many core nucleosome components and chromatin modifiers (Fig. 4c). Most of these pathways were equally represented among cold- and heat-responsive genes. Cytoskeletal components, however, were predominantly associated with cold temperature, while chromatin and histone components were predominantly associated with warm temperature.

We confirmed these results using automated GO-term analysis. This approach identified similar key terms and pathways in the data set (Fig. 4d, Additional file 8: Table S3). ORFs that could not be assigned a conserved function also contained genes of interest. For example, two ORFs (NEME_164146 and NEME_174994) with negative loadings for PC2 coded for stonustoxin- and neoverrucotoxin-related proteins. Other temperature-responsive ORFs (such as NEME_198370 or NEME_70247) were conserved among fish.

## Discussion

Our analyses support our a-priori assumption that zygotic transcription starts after the 32-cell stage in the round goby. We found that cleavage stage correlates with expression only at baseline levels (as do genetic similarity and temperature interval experienced by the mother). This indicates that maternal RNA is stable across the developmental stages analysed. This is to be expected for maternal RNA, which is mostly stable up to the Maternal-Zygotic Transition, and degraded in a tightly controlled process through targeted decapping and deadenylation ([1, 17, 26, 41]. Due to its default state of stability, maternal RNA helped identify and characterize many of the regulators of mRNA stability we know today. If any of our samples would have passed the MZT, we would expect a massive signature of transcriptome turnover concomitant with cleavage progression. Cleavage stage would correlate with expression levels more than any other parameter, and cleavage stage would relate to the major principal components. Neither of those is the case (Figs. 2 and 3), which confirms that MZT happens after the 32-cell stage in the round goby. Since we cannot block RNA degradation in our system, we cannot, however, rule out the possibility that maternally directed RNA degradation systems (which act at the same time but are independemt from the MZT; [41]) are sensitive to seasonally variable parameters and therefore contribute to the observed effects.

Our results show a link between the expression levels of many maternally contributed genes and the maternal temperature experience. In the context of fish ecology, temperature is a proxy for a range of seasonal variations, such as light intensity or seasonal abundance of specific prey items. Accordingly, the observed effects may be related to any seasonally fluctuating parameter. Parental diet, for example, has been shown to affect the composition of maternal RNA in zebrafish [44]. Importantly, egg quality is not linked to season in the round goby. Egg quality is defined as the ability of the embryo to develop to hatching and has been linked to female size, genetics, handling, stress, non-optimal timing of oocyte harvest, induced ovulation, yolk composition, protein and hormone content, and RNA content of oocytes ([10, 8, 68] and references therein). Round goby hatching success is very high (~95% [13]) and stable throughout the year ([28]; own observation). Also, resources are not limiting at the sampling site. Food (mussels and gammarids) is abundant, and females have identical length-weight ratios in the early, late, and outside the spawning season (own observation). In fact, round goby withhold eggs rather than produce a poor quality batch in suboptimal conditions [29]. Therefore, available data do not support an interpretation of the observed effects by a general deterioration of egg quality or female condition along the season.

Our findings imply that wild species are able to adapt the levels of maternally contributed RNA messages (within developmental constraints) to key environmental factors. We expect that this is particularly true for fish, amphibians, or birds. Those species rely more on maternally contributed factors for embryonic development than others [59]. Mammals are generally less dependent on maternally contributed mRNA. Nonetheless, they require maternal genes for development [36]. Mice embryos start to express their own genes only at the 2- to 4-cell stage [64], human embryos at the 4- to 8-cell stage [9]. In mammals, RNAs paternally contributed through sperm have recently been reported to mediate parental experiences under laboratory conditions [23, 52], and may also be relevant for non-genetic inheritance under natural conditions.

Many pathways and molecules have been suggested to carry information from one generation to the next. For example, hormones or proteins can be allocated in varying amounts into the egg by the mother, as has been shown for androgen levels in collared flycatchers [51]. Also, the extent of maternal provisioning with energy can affect offspring fitness. For example, the amount of fatty acids provided in the egg has been shown to impact the performance of marine fish offspring [21]. Epigenetic mechanisms such as DNA methylation have also been suggested to contribute to transgenerational information transfer in wild vertebrates [65]. Our results suggest for the first time that the maternal RNA contribution may also present a relevant mechanism. In stickleback and tilapia, temperature tolerance has a strong maternal component [45, 56]. Possibly, maternally provided RNAs may contribute to the observed intergenerational memory of maternal temperature experience. Whether or not many species use maternal RNA to inherit temperature information remains speculation. Based on the idea that “nothing in biology makes sense except in the light of evolution” [15], we propose that evolution will favor the use of any biological pathway which has the capacity to enhance the performance and the fitness of individuals and species. Accordingly, we speculate that each species will use the pathway which is most suited to transmit the most relevant information in its specific environment.

It remains to be determined whether the observed changes in expression levels have any information value for round goby offspring. Certainly, functional annotations can only suggest, but not predict, possible outcomes for offspring. Human orthologues, which are used in most cases to determine gene roles, may have functionally diverged from their round goby counterparts. Also, ORFs for which we could not infer a function may be highly relevant for temperature pre-adaptation. However, the identified gene groups and pathways allow to develop testable hypotheses on intergenerational priming strategies of wild vertebrates. For example, one may speculate that fish react to temperature or stressors by varying histone expression and global chromatin dynamics in their offspring as an epigenetic diversification strategy. Introducing a random component to gene expression may enable offspring to follow a diverse range of reaction norms or developmental trajectories without the need for genetic variation in temperature-related genes. Also, the fluidity and dynamics of biological membranes depend on ambient temperature. Adjusting lipid metabolism, lipid modification, and cell adhesion during morphogenesis in dependence of environmental conditions seems like a reasonable approach for a poikilotherm organism. Finally, many of the identified pathways are associated with signaling and transcriptional regulation. Regulatory pathways have the capacity to amplify and generate a more pronounced downstream effect during embryogenesis. It has been pointed out previously that small changes in expression have big effects when they feed into gene regulatory networks [14]. Also, highly expressed genes or genes that undergo large changes in expression are not necessarily the best predictors of complex phenotypic traits. For example, minute variations (< 0.2 fold) in the ovarian expression levels of > 250 genes together explained > 90% of embryo survival in striped bass, while no single gene accounted for more than 2% of the effect [12]. Thus, minor changes in the levels of maternally contributed transcriptional regulators and signaling pathways may be able to generate large effects at a later timepoint in embryonic development.

To clarify the functional relevance of individual pathways, exposure and manipulation experiments will be required, for example using antisense or mRNA injections on oocytes and embryos. Screening 3′ and 5′ regulatory gene regions of temperature-sensitive genes for conserved sequence motifs may allow us to gain a mechanistic understanding how maternal environmental perception and gene expression regulation are coupled during oogenesis. Finally, two major questions remain to be answered in the future: Which types of parental experiences affect the composition of maternal RNA contribution? and: Would different experiences cause different change patterns, or would they converge on similar sets of genes to more generally enhance plasticity?

## Conclusions

In conclusion, wild vertebrate populations may rely on non-genetic means to increase their survival chances more than we appreciate today. We may not be aware of many mechanisms simply because they have not been investigated in the appropriate natural settings and in non-model organisms. From this follows that, if the maternal RNA contribution (or other parental contributions, such as proteins, hormones, and epigenetic marks) can be fine-tuned (as suggested by our experiments), such an adjustment may potentially contribute to the success (or failure) of adaptation processes [58]. Accordingly, the ability to provide non-genetic information may be a feature under selection itself. We propose that the superior success of species such as the invasive round goby in changing environments may be attributable to a superior ability of those species to inform and pre-adapt their offspring. Finally, our approach validates that wild vertebrate populations which comply with evolutionary constraints of parental effects [33] may be an excellent place to gain deeper insights into mechanistic aspects of parental effects.

## Methods

### Collection of embryos from the wild

Embryos were sampled between May and August 2015 in the harbor Kleinhüningen in Basel, Switzerland (47.587448 N, 7.593409 E). This is a cargo harbor comprising two basins, with approximately 4500 incoming ships per year, in the river Rhine [3]. Eight spawning traps containing PVC tubes in a basket were built and were deployed with cables to bottom of the second, more remote basin, which is connected to the river only through a 12 m wide and 129 m long passage and the 176 m wide first basin. Traps were deployed at 4 m depth according to Hirsch et al. [28], raised twice a week, and PVC tubes opened and inspected for eggs. Typically, one tube would contain between 250 and 2000 eggs laid by 1 to 4 females, with clutches from different females discernible by egg color variations between pale yellow and orange, arranged in circular patterns around the initial clutch. Samples were taken only when the arrangement of eggs and colors allowed to unequivocally distinguish between clutches laid by different females, or when only a single small clutch was present. Eggs were picked from the PVC tubes with a forceps and distributed to three Eppendorf tubes. The first sample was directly picked into 100% Ethanol in the field, transported on crushed ice, and stored at 4 °C in the laboratory, to be used for used for species confirmation by barcoding. The second sample was directly picked into fixative (4% methanol free formaldehyde; Formaldehyde 30% methanol free (ROTH #4235.1) diluted with 1 x PBS) in the field, transported on crushed ice, and stored at room temperature, to be used for cleavage stage identification. The third sample was picked into an empty tube in the field, flash frozen in liquid nitrogen on site, transported in liquid nitrogen, and stored at − 80 °C, to be used for RNA sequencing. There were no discernible differences between eggs laid early or late in the season in terms of morphology or size.

### Temperature parameters

Water temperature was recorded at the site of embryo collection from April to August every 30 min using a Dissolved Oxygen Logger (HOBO U26–001) which was attached to one of the spawning traps and located at 4 m depth. From the recordings, average, median, minimum and maximum temperature in the five days preceding sampling, as well as the temperature interval in that time frame, were calculated.

### Species confirmation by genotyping

At the time of sampling, two related Ponto-Caspian goby species, bighead goby (*Ponticola kessleri*) and round goby (*Neogobius melanostomus*), were present at the sampling site, although the latter were much more abundant than the former. The two species produce very similar eggs that are difficult to distinguish visually. Therefore, the species identity of each sample was confirmed by genotyping. DNA was extracted from the embryos using the DNeasy Blood and Tissue kit (Qiagen). Samples were genotyped using primers NG236.1 f (CAGGCTGAACCATATGGCAG, [3]), NG236 r (AGATCCTCCCCAACCAAGAT, [61]), Nme 7 f (AATGGATGGGTCAATTGCAT) and Nme 7 r (AAGGTTGAGCTGCCACTGAG; both [16]) and FastStart Taq DNA Polymerase (Roche). All four primers were used in the same PCR reaction. The primer pair NG236.1f / NG236r is specific to *P. kessleri* and amplifies a 150 bp fragment, while the primer pair Nme7f / Nme7r is specific to *N. melanostomus* and amplifies a 300 bp fragment. All sampled clutches were confirmed to be *N. melanostomus*.

### Identification of early cleavage embryos

Within three days after sampling, 10–15 embryos from each sample were dechorionated in 1× PBS, permeabilized in PBST (1 x PBS + 0.1% Triton X-100) at 4 °C over night, and stained with Phalloidin (1:1000 dilution of Fluorescent Dye 488-I Phalloidin (LucernaChem, #U0281) in PBST + 1% BSA) for 2 h at room temperature. After two washes in PBST, embryos were embedded on glass slides equipped with two CoverWell silicone isolators (Electron Microscopy Sciences) in Fluoromount G (Southern Biotech). Developmental stage was assessed under a Nikon E400 Eclipse microscope according to Kimmel [31].

### RNA extraction

Based on the staging, fourteen samples that had not yet progressed past the 32-cell stage were chosen for sequencing (Fig. 1c, Table 3). Additionally, one sample that had progressed past the somite stage was included as internal reference (Table 3). RNA was extracted from ~ 1 ml (20–30) embryos using a standard phenol-chloroform protocol combined with a Single Cell RNA Purification Kit (Norgen, # 51800). Embryos were ground in 1.5 ml Trizol in liquid N_2_. The resulting powder was transferred into tubes (Eppendorf), and the homogenate was centrifuged for 10′ at 12000 g at 4 °C to separate lipids. The liquid portion of the sample was transferred to a new tube. 0.2 ml chloroform were added, the sample was vortexed for 15″, incubated for 5′ at room temperature, and again centrifuged for 15′ at 12000 g at 4 °C. 250 μl of the resulting supernatant were combined with 250 μl Buffer RL from the Single Cell RNA Purification Kit and 700 μl EtOH for further processing. Further steps were proceeded according to the manufacturer’s instruction of the Single Cell RNA Purification Kit. RNA was eluted in two steps with 8 μl and 20 μl to optimize yield and volume. DNA was removed from the sample with an RNase-Free DNase I Kit (Norgen, # 25710) according to the manufacturer’s instructions.

**Table 3:** Sample specifications

			Water temperature on 5 days before sampling day (°C)											
Sample number	Sampling date	Developmental stage	Mean	Maximum	Minimum	Median	Interval	Mean water temperature on sampling day (°C)	Sequencing library	Individual dissimilarity	Raw read pairs	QF read pairs	% retained	% total
2	15-May-15	8-cell stage	15.30	16.56	13.94	15.20	2.62	16.06	1	0.865	23,797,396	23,679,970	99.5	5.3
50	1-Jun-15	1 cell stage	15.54	16.38	14.48	15.63	1.90	16.49	1	0.805	47,705,400	47,500,770	99.6	10.7
28	22-May-15	4-cell stage	15.58	16.28	14.76	15.64	1.52	14.84	2	0.915	20,373,120	19,616,406	96.3	4.4
30	22-May-15	32-cell stage	15.58	16.28	14.76	15.64	1.52	14.84	1	0.76	26,604,709	26,292,764	98.8	5.9
70	3-Jun-15	16–32 cell stage	16.19	17.28	15.26	16.10	2.02	17.55	2	1.106	40,054,067	39,820,682	99.4	8.9
80	8-Jun-15	16–32 cell stage	17.00	18.68	15.72	16.90	2.96	19.01	2	0.9	50,809,855	50,559,355	99.5	11.4
132	26-Jun-15	4–8 cell stage	18.25	19.22	17.50	18.24	1.72	18.80	1	0.83	16,474,441	15,903,806	96.5	3.6
135	26-Jun-15	1-cell stage	18.25	19.22	17.50	18.24	1.72	18.80	1	1.165	31,063,597	30,915,991	99.5	6.9
98	5-Jun-15	1–2 cell stage	19.01	21.46	17.14	18.96	4.32	20.70	2	0.785	49,901,005	49,640,357	99.5	11.2
123	22-Jun-15	16-cell stage	19.03	19.74	18.22	19.08	1.52	18.35	2	1.015	21,550,669	21,364,189	99.1	4.8
127	22-Jun-15	2-cell stage	19.03	19.74	18.22	19.08	1.52	18.35	2	1.095	20,752,958	20,628,391	99.4	4.6
147	3-Jul-15	1-cell stage	20.66	23.06	19.42	20.38	3.64	22.54	1	1.12	20,273,141	20,149,106	99.4	4.5
148	3-Jul-15	16-cell stage	20.66	23.06	19.42	20.38	3.64	22.54	1	1.105	40,050,217	39,840,710	99.5	9.0
167	10-Jul-15	4-cell stage	23.95	24.60	23.30	23.98	1.30	23.16	2	0.76	24,849,517	24,685,544	99.3	5.5
141	3-Jul-15	prim stage	20.66	23.06	19.42	20.38	3.64	22.54	1	NA	14,498,531	14,346,106	98.9	3.2

### Library preparation and sequencing

Maternal RNA is heavily regulated at the post-transcriptional stage [66], which implies that maternally contributed mRNAs are not necessarily polyadenylated [47]. We therefore used the Ribo Zero Gold rRNA Removal Kit to deplete ribosomal RNAs, and random hexamer primers instead of poly-T primers for reverse transcription, to avoid bias against deadenylated mRNA species. Two sequencing libraries were prepared from 1 to 2 μg of RNA per sample at the Genomics Facility in Basel (https://www.bsse.ethz.ch/genomicsbasel). Samples were paired-end sequenced on an Illumina NextSeq 500 using 81 cycles. We obtained a total number of 448′758’623 reads, which distributed to individual samples as indicated in Table 3. Downstream data analysis was performed using the HPC Cluster sciCORE (http://scicore.unibas.ch/) of the scientific computing core facility at University of Basel.

### Assembly of the transcriptome

In a first step, raw reads were filtered according to the following parameters using Prinseq-lite (V0.20.4, [53]): min length 75, mean quality 15, no ambiguous nucleotides, low complexity filter dust with threshold 10. Filtered reads were then de-novo assembled with Trinity (Version 2.1.1, [25]) using the following parameters: paired-end assembly, min contig length 100, min glue 4, group pairs distance 300, kmer cov 4. ORFs were identified with Transdecoder as part of Trinity using the following parameters: min protein length 50. In order to remove redundancy, ORFs were de-replicated and clustered using Usearch (V8.1.1812, [19]) with the following parameters: remove duplicates, cluster coverage 100%, identity 98%. The transcriptome was then cleaned of rRNA using Blast+ (Version 2.2.31) and Silva LSU and SSU reference databases V123 with the following parameters: e-value = 0.001. These steps resulted in an embryonic transcriptome of *N. melanostomus* of 101,476 ORFs. A count table was generated using BWA mem (V0.7.12) for mapping, SAMtools (V1.2) to sort the sam file, and SAM2counts (https://github.com/vsbuffalo/sam2counts) to get read counts per ORF.

### Cleaning and validation of the transcriptome

Before downstream analysis, ORFs derived from transposable elements, ORFs not matching gene models, and ORFs with very low count numbers were removed to reduce noise. The assembled ORFs were screened for transposable elements using RepeatMasker (Version 4.0.6, http://www.repeatmasker.org/) using the zebrafish reference database. 4977 (5%) potentially TE derived ORFs were removed from the transcriptome. ORFs were then matched with gene models obtained from an automated annotation (based on Augustus and Maker, Tomas Larsson, University of Gothenburg, unpublished data) of the round goby genome sequence (Irene Adrian-Kalchhauser, unpublished data). 46,560 of all assembled ORFs (46%) matched gene models. In comparison, non-matching ORFs had lower count numbers, were on average shorter than matching ORFs, and had few blast matches in existing databases. Since their identity and function could not be specified, they were omitted from further analysis. Before normalization, ORFs with very low counts (2 or less per ORF; 25% of the data) were removed. These ORFs were predominantly expressed at later stages (in sample A141) but not during early cleavages (all other samples). The remaining 35,037 ORFs were normalized using DESeq2 ([37]; table of non-normalized counts: Additional file 5: Table S2).

We then compared our transcriptome with maternally provided messages identified in zebrafish embryos by Rauwerda et al. [49]. Using Ensembl Biomart [4], we retrieved and blast searched coding sequences considered expressed (*n* = 11,118) and non-expressed (*n* = 11,353) in zebrafish embryos by Rauwerda et al. [49] against our transcriptome. This approach yielded 1617 and 782 round goby ORF orthologues, respectively, for which we plotted median expression values.

### SNP calling and calculation of individual dissimilarity

Reads2snp (Version 2.0, [24]) was used to identify possible SNPs in the round goby tanscriptome. SNPs below a minimum number of 30 reads, minimum base quality of 20, and minimum mapping quality of 10 were not recorded. Vcftools (Version 0.1.14) was used to filter the SNP table and sites with missing data for any of the 14 samples were removed. These criteria resulted in a total of 4′096 SNPs that were clustered. Individual dissimilarity was calculated using the Bioconductor package SNPrelate [70].

### Principal component analysis and correlation analysis

Principal component analysis (PCA) was performed with prcomp from the R base package on normalized ORF expression values using mean temperature, temperature interval, cleavage stage, and individual dissimilarity as explanatory variables. The same parameters were used to calculate rank-based Spearman correlation values with the expression values of each ORF in R.

### Functional annotation

For functional annotation, open reading frames which would display a two-fold or higher change in expression in response to temperature, and where Spearman correlation values with temperature would exceed ±0.7, as well as open reading frames that loaded above average on the PC analysis, were batch blasted using Blast2Go Basic (www.blast2go.com). The behavior of those open reading frames is visualized in biplots in Additional file 4: Data S1. Gene descriptions returned by Blast2Go were used to identify ortholog human genes. If Blast2Go did not return results, ORFs was manually blasted using blastx (https://blast.ncbi.nlm.nih.gov/Blast.cgi). For manual annotation, we mined Uniprot and NCBI gene descriptions. For automated cluster analysis with DAVID [30], human Ensembl IDs were retrieved from Ensembl BioMart. As background gene list, genes considered expressed by Rauwerda et al. [49] were translated into human Ensembl IDs. Functional clustering was based on FAT GO terms, cluster affinity was set to “high”. For the figure, similar clusters (such as “negative regulation of transcription” and “positive regulation of transcription”) were summarized (for example as “transcriptional regulation”), and the highest enrichment score of all clusters feeding into the summary category was retained.

## Additional files

Additional file 1: **Figure S1.** Embryonic development of *N. melanostomus*. Phalloidin stainings of embryos collected in the field, ordered by developmental stage. (PDF 14950 kb)

Additional file 2: **Table S1.** Read counts. The table contains raw read counts for all ORFs that match a predicted open reading frame according to AUGUSTUS and MAKER annotations of the round goby draft genome. (TXT 2672 kb)

Additional file 3: **Data S1.** The *N. melanostomus* maternal transcriptome. FASTA sequences of de-novo assembled, maternally expressed open reading frames which match gene models in the draft N. melanostomus genome. (PDF 2139 kb)

Additional file 4: **Figure S2.** Biplots of control and testing variables. (PDF 157 kb)

Additional file 5: **Figure S3.** PCA correlations. (PDF 137 kb)

Additional file 6: **Data S2.** Supplementary analyses of RNA sequencing data. Biplots between samples, biplots with correlating genes or PCA loading genes marked, comparison of correlation values and PCA loading. (PDF 1792 kb)

Additional file 7: **Table S2.** Functional annotation. (XLSX 76 kb)

Additional file 8: **Table S3.** DAVID GO term analysis. (XLSX 110 kb)
