# Supplementary figures and images for: RNA sequencing of early round goby embryos reveals that maternal experiences can shape the maternal RNA contribution in a wild vertebrate
Source: BMC Evol Biol. 2018 Mar 22;18:34. doi: 10.1186/s12862-018-1132-2 (PMC5863367; doi:10.1186/s12862-018-1132-2)

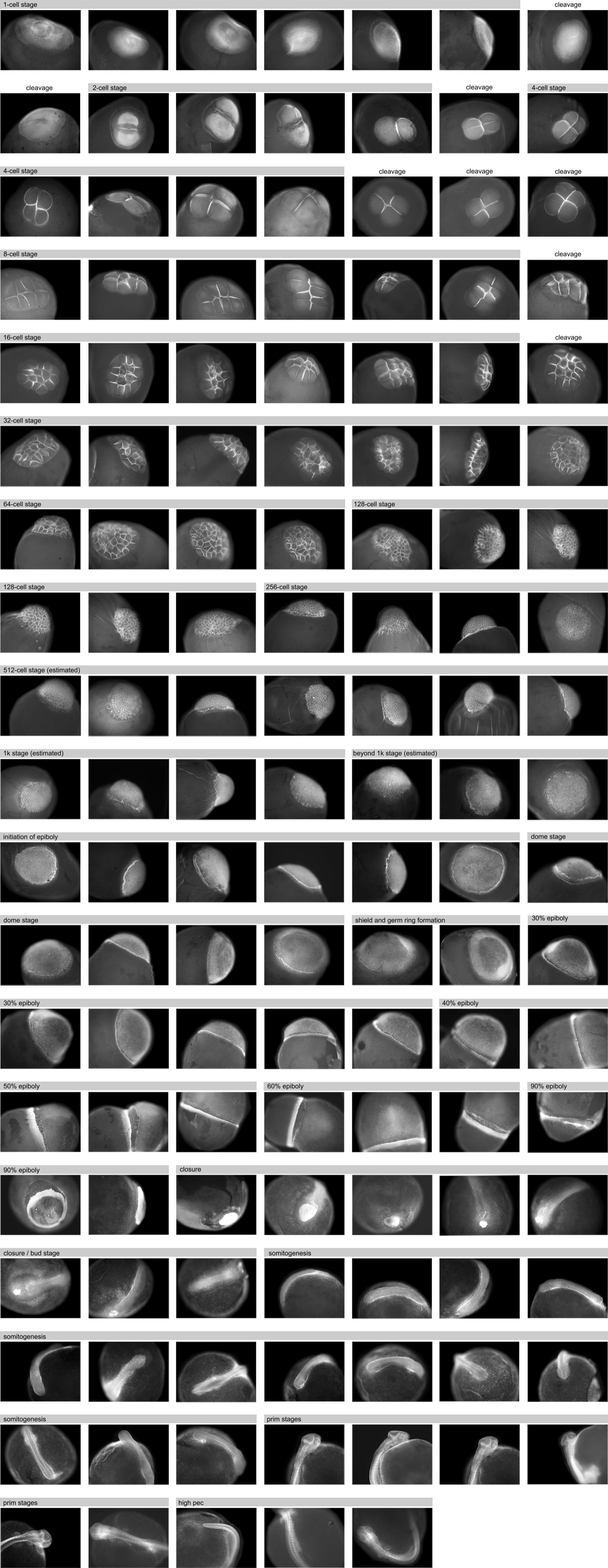

Supplement: Supplementary file 1 — Figure S1. Embryonic development of N. melanostomus. Phalloidin stainings of embryos collected in the field, ordered by developmental stage. (PDF 14950 kb) [file 12862_2018_1132_MOESM1_ESM.pdf]

Adrian-Kalchhauser et al.  
Figure S3

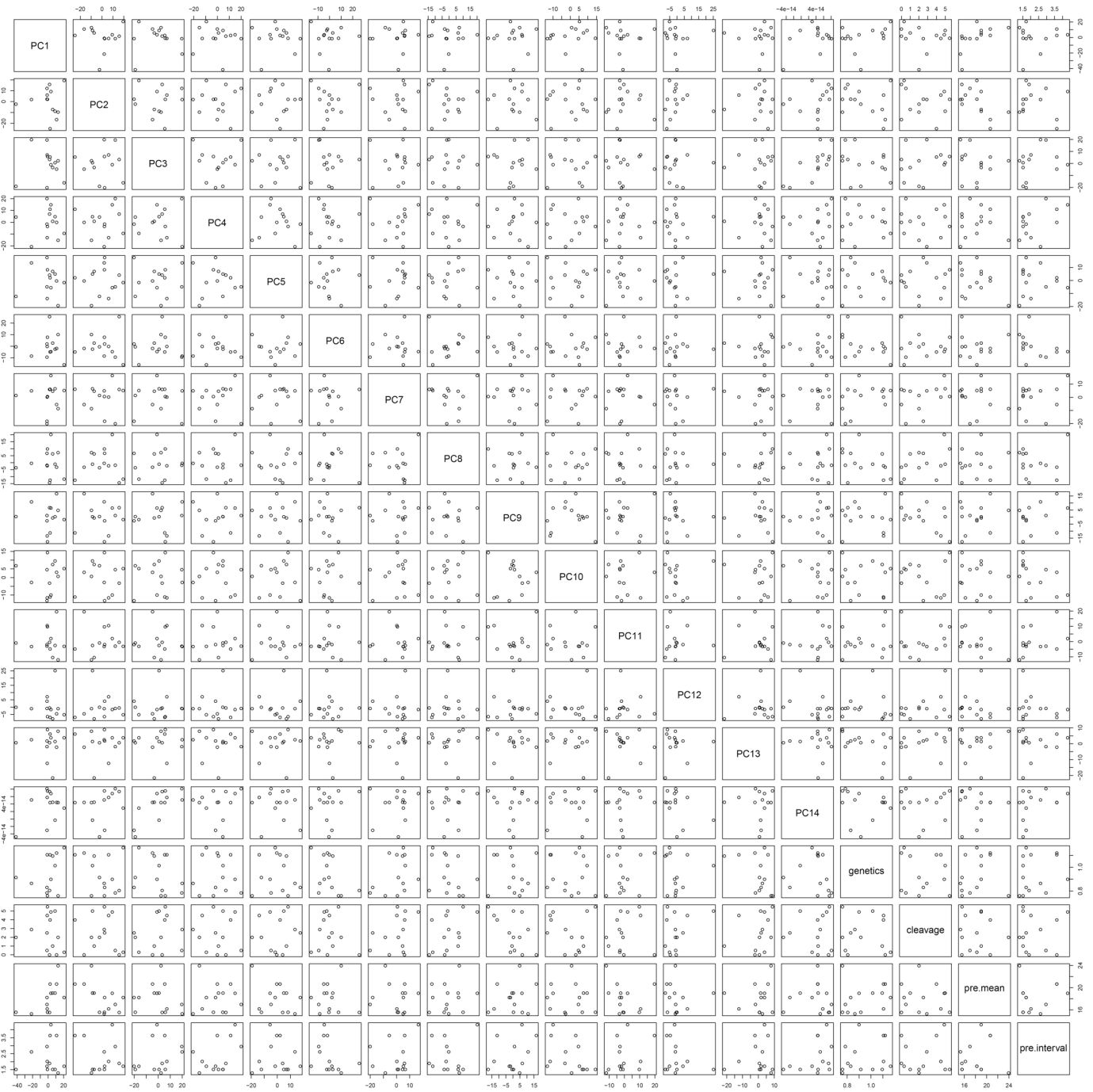

Supplement: Supplementary file 3 — Data S1. The N. melanostomus maternal transcriptome. FASTA sequences of de-novo assembled, maternally expressed open reading frames which match gene models in the draft N. melanostomus genome. (PDF 2139 kb) [file 12862_2018_1132_MOESM3_ESM.pdf]
