# Supplementary material for: RNA sequencing of early round goby embryos reveals that maternal experiences can shape the maternal RNA contribution in a wild vertebrate
Source: BMC Evol Biol. 2018 Mar 22;18:34. doi: 10.1186/s12862-018-1132-2 (PMC5863367; doi:10.1186/s12862-018-1132-2)

Figure S2. Parameters used for correlation analysis.

RNA sequencing of early round goby embryos reveals that maternal experiences can shape the maternal RNA contribution in a wild vertebrate

Irene Adrian-Kalchhauser, Jean-Claude Walser, Michaela Schwaiger, Patricia Burkhardt-Holm

“Pre-mean”/”Pre-min”/”Pre-max”/”Pre-median” = mean/minimum/maximum/median temperature experienced by the mother in the last five days before oviposition.

“Pre-interval” = temperature interval experienced by the mother in the last five days before oviposition.

“Day-mean”/”Day-min”/”Day-max” = mean/minimum/maximum temperature recorded on the sampling day.

“Cleavage” = number of completed cleavage divisions.

“Genetics” = genetic dissimilarity of the samples.

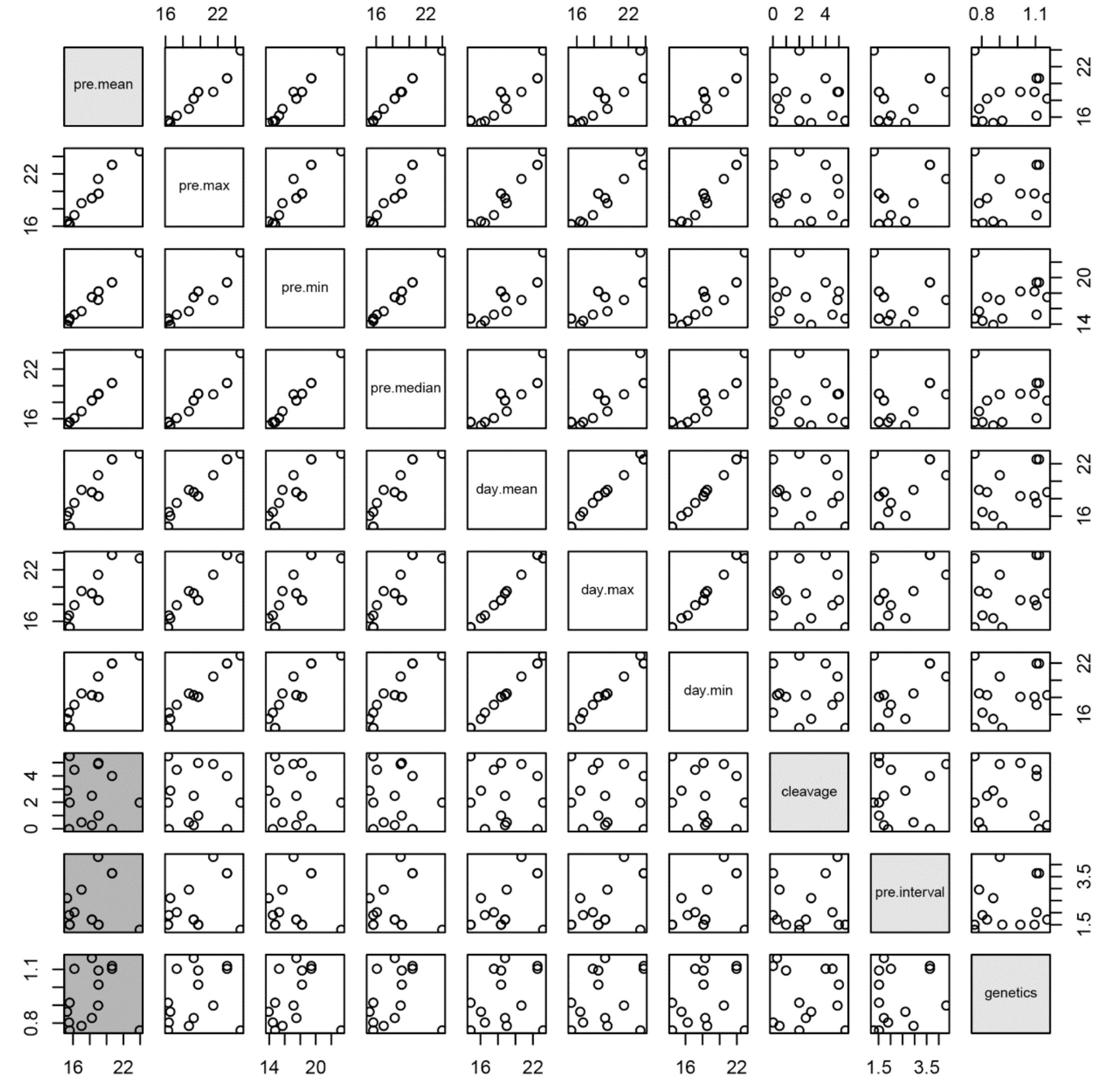

Supplement: Supplementary file 4 — Figure S2. Biplots of control and testing variables. (PDF 157 kb) [file 12862_2018_1132_MOESM4_ESM.pdf]
