# Supplementary material for: RNA sequencing of early round goby embryos reveals that maternal experiences can shape the maternal RNA contribution in a wild vertebrate
Source: BMC Evol Biol. 2018 Mar 22;18:34. doi: 10.1186/s12862-018-1132-2 (PMC5863367; doi:10.1186/s12862-018-1132-2)

Figure S3. PCA correlations.

RNA sequencing of early round goby embryos reveals that maternal experiences can shape the maternal RNA contribution in a wild vertebrate

Irene Adrian-Kalchhauser, Jean-Claude Walser, Michaela Schwaiger, Patricia Burkhardt-Holm

Correlation plot of all Principal Components with each other and with cleavage, mean temperature experienced by the mother, and temperature interval experienced by the mother.

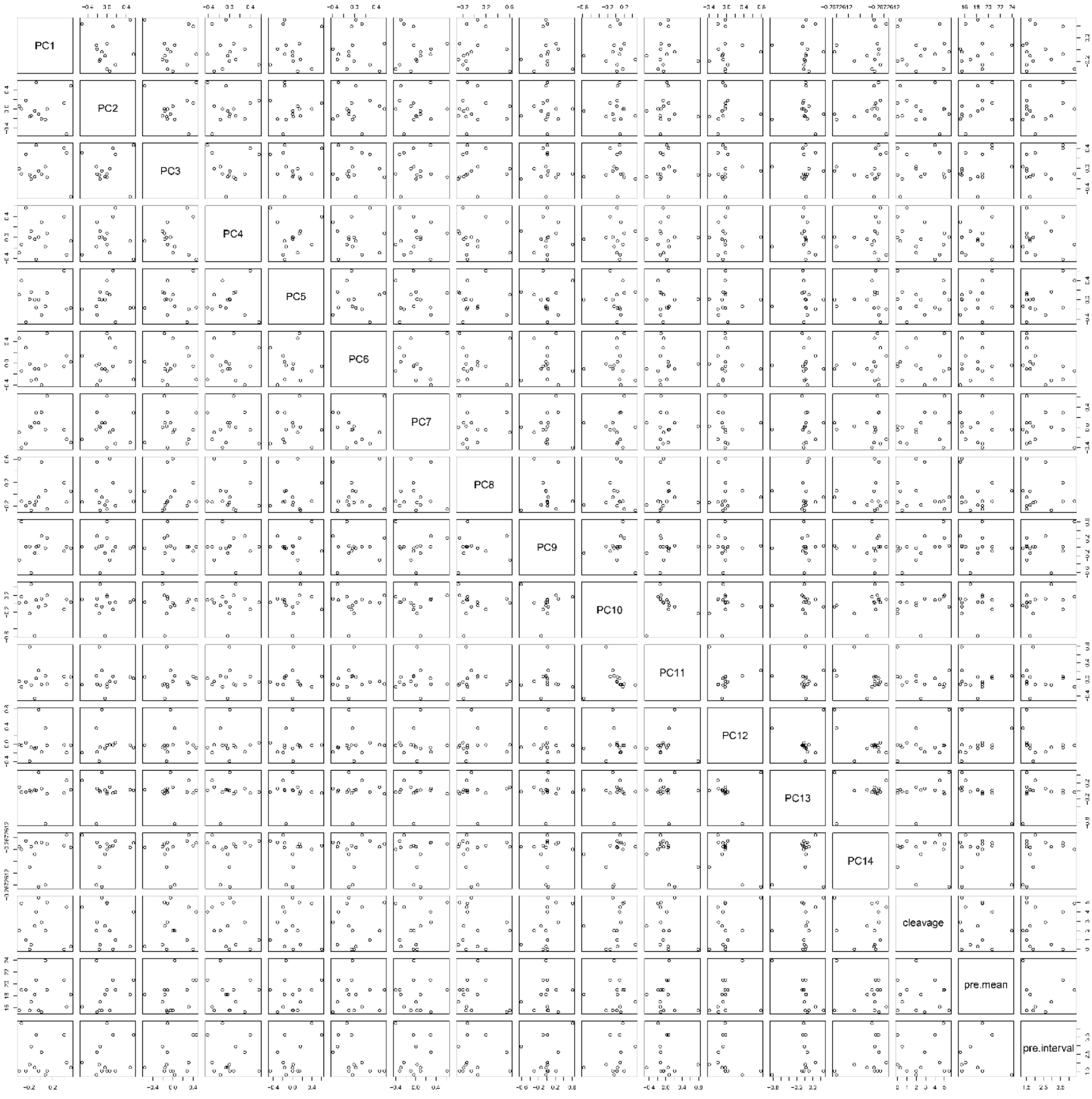

Supplement: Supplementary file 5 — Figure S3. PCA correlations. (PDF 137 kb) [file 12862_2018_1132_MOESM5_ESM.pdf]
