# Supplementary material for: RNA sequencing of early round goby embryos reveals that maternal experiences can shape the maternal RNA contribution in a wild vertebrate
Source: BMC Evol Biol. 2018 Mar 22;18:34. doi: 10.1186/s12862-018-1132-2 (PMC5863367; doi:10.1186/s12862-018-1132-2)

**Data S2. Supplementary analyses of RNA sequencing data.**

**RNA sequencing of early round goby embryos reveals that maternal experiences can shape the maternal RNA contribution in a wild vertebrate**

Irene Adrian-Kalchhauser, Jean-Claude Walser, Michaela Schwaiger, Patricia Burkhardt-Holm

## 1. Biplots of normalized count data between samples.

Shown are log2 expression values after normalization with DESeq2.

The density of data in each sample from low to high expression is indicated by histograms above the biplots.

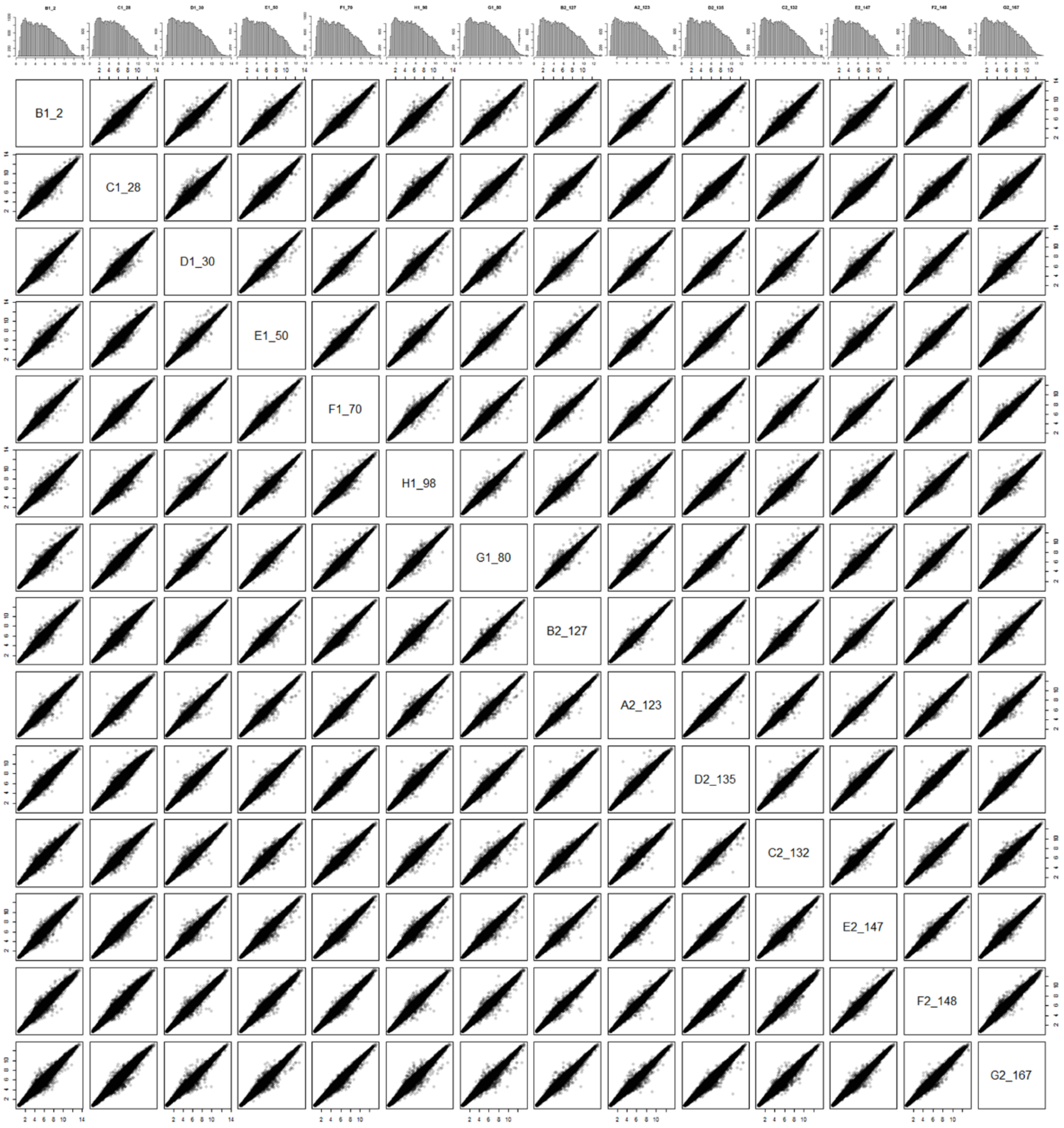

## 2. Behavior of highly correlating and anticorrelating open reading frames

Top: Biplots between expression values in the indicated sample (horizontal axis) and median expression values in the data set (vertical axis). Samples are ordered from lowest to highest median temperature before oviposition. Bottom: Biplot between expression in the “coldest” and “warmest” sample.

In each plot, those open reading frames that are correlating and anticorrelating with mean maternally experienced temperature (r >= 0.7 and <= -0.7) and display a 2x fold change between the minimum and maximum expression value are indicated in green and red, respectively.

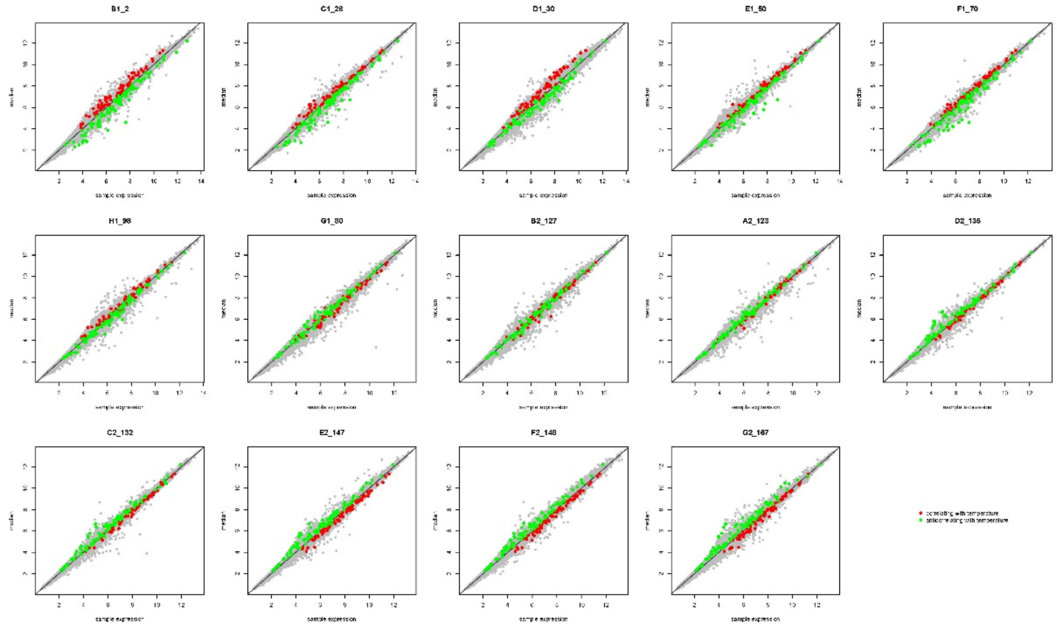


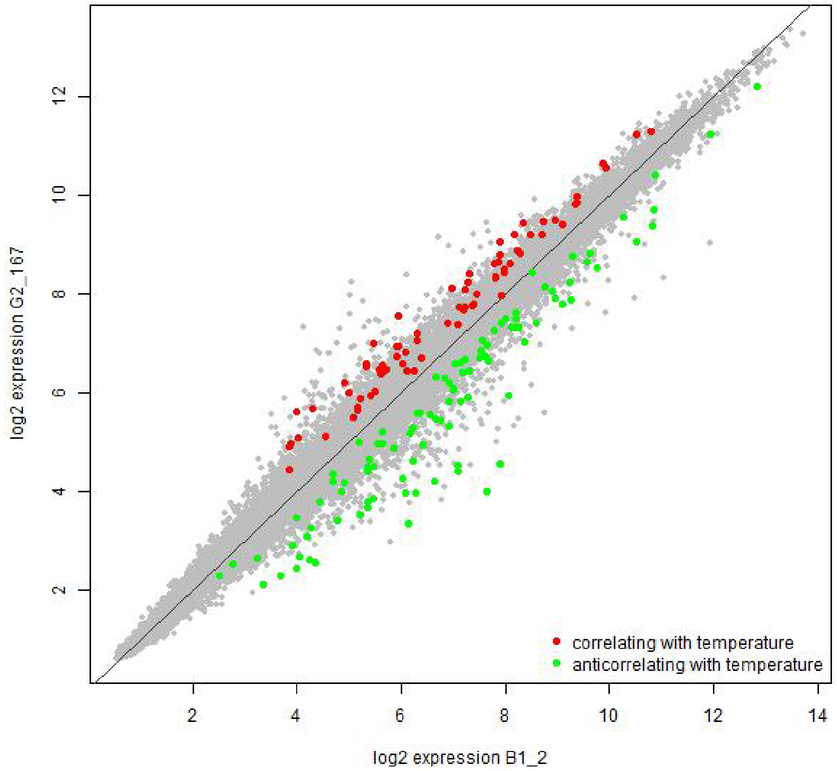

## 3. Behavior of open reading frames loading in PC1 and PC2

Top: Biplots between expression values in the indicated sample (horizontal axis) and median expression values in the data set (vertical axis). Samples are ordered from lowest to highest median temperature before oviposition. Bottom: Biplot between expression in the "coldest" and "warmest" sample. In each graph, the 20 top and bottom loading genes on PC1 and PC2 (see legend for color code) are indicated with green and red dots.

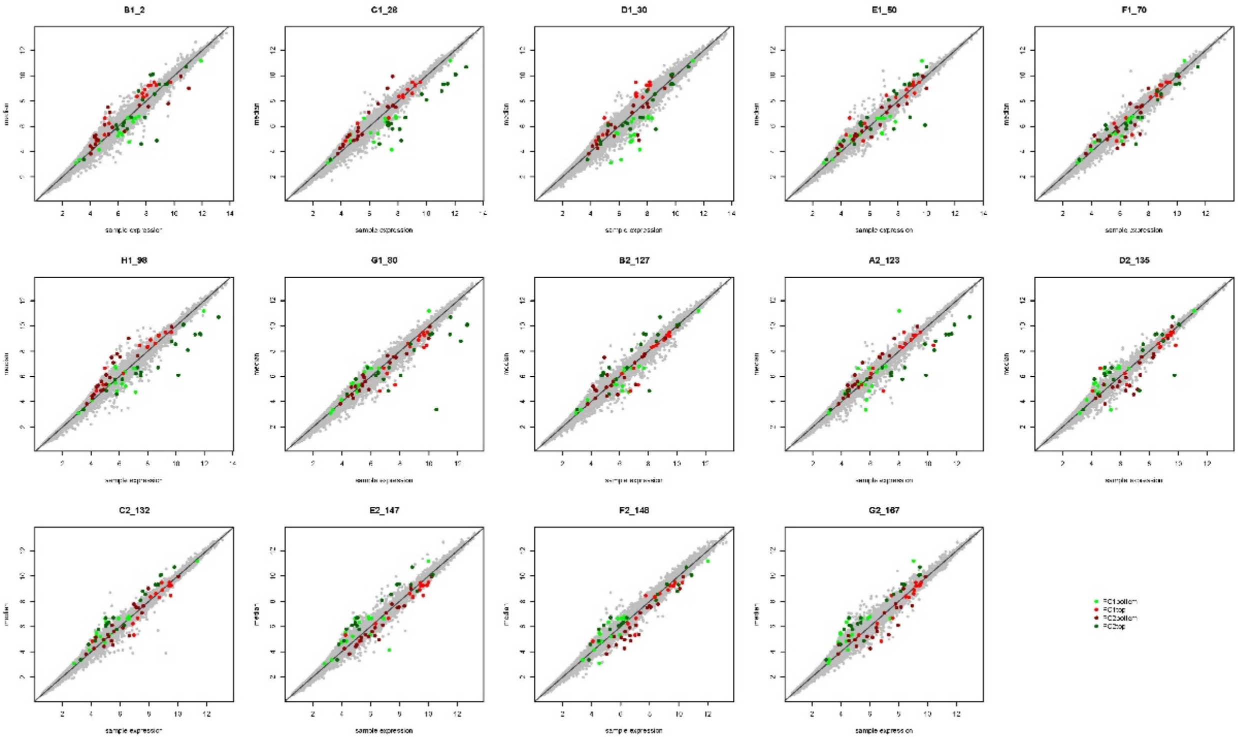


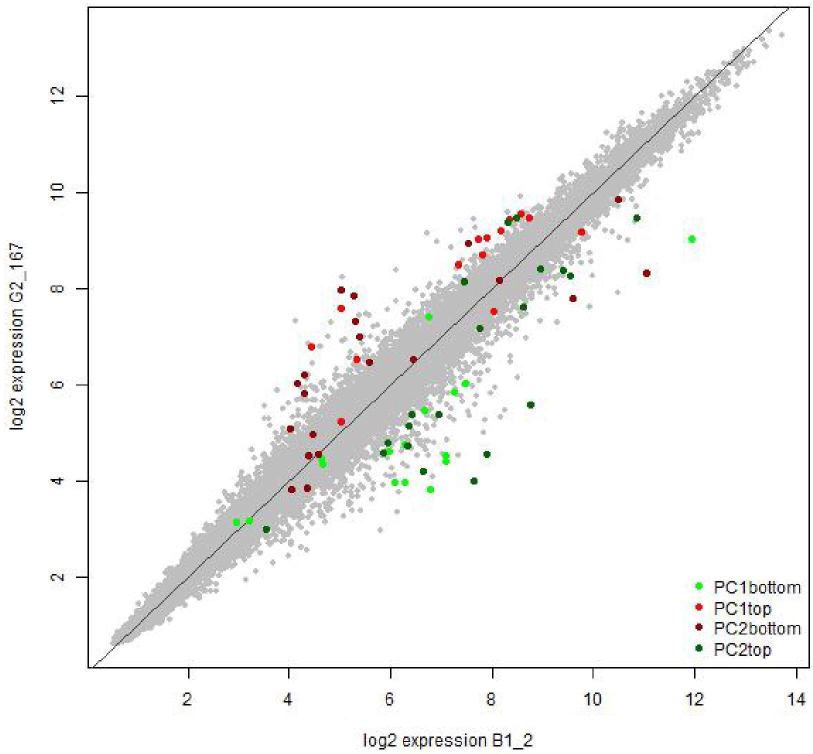

## 4. Comparison of PC loading values and correlation values

PC1 and PC2 loadings (vertical axis) are plotted against correlation values (horizontal axis). The two approaches identify an overlapping group of open reading frames, even though the rank of many open reading frames in PC loading and in correlation analyses differs.

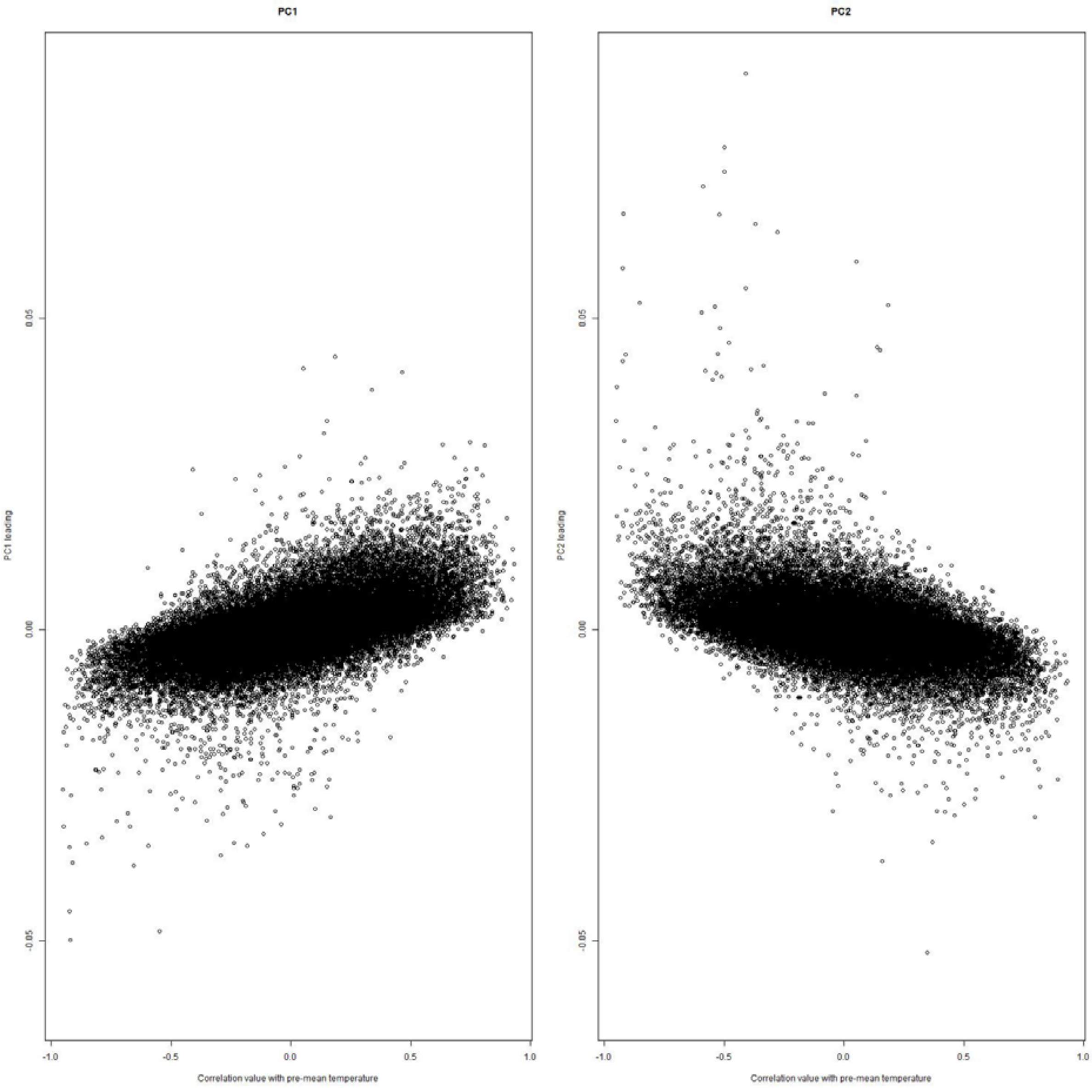

Supplement: Supplementary file 6 — Data S2. Supplementary analyses of RNA sequencing data. Biplots between samples, biplots with correlating genes or PCA loading genes marked, comparison of correlation values and PCA loading. (PDF 1792 kb) [file 12862_2018_1132_MOESM6_ESM.pdf]
